# *Batf3*-Dependent CD11b^low/−^ Peripheral Dendritic Cells Are GM-CSF-Independent and Are Not Required for Th Cell Priming after Subcutaneous Immunization

**DOI:** 10.1371/journal.pone.0025660

**Published:** 2011-10-17

**Authors:** Brian T. Edelson, Tara R. Bradstreet, Wumesh KC, Kai Hildner, Jeremy W. Herzog, Julia Sim, John H. Russell, Theresa L. Murphy, Emil R. Unanue, Kenneth M. Murphy

**Affiliations:** 1 Department of Pathology and Immunology, Washington University School of Medicine, St. Louis, Missouri, United States of America; 2 Howard Hughes Medical Institute, Washington University School of Medicine, St. Louis, Missouri, United States of America; 3 Department of Developmental Biology, Washington University School of Medicine, St. Louis, Missouri, United States of America; University of Crete, Greece

## Abstract

Dendritic cells (DCs) subsets differ in precursor cell of origin, functional properties, requirements for growth factors, and dependence on transcription factors. Lymphoid-tissue resident CD8α^+^ conventional DCs (cDCs) and CD11b^low/−^CD103^+^ non-lymphoid DCs are developmentally related, each being dependent on FMS-like tyrosine kinase 3 ligand (Flt3L), and requiring the transcription factors *Batf3*, *Irf8*, and *Id2* for development. It was recently suggested that granulocyte/macrophage colony stimulating factor (GM-CSF) was required for the development of dermal CD11b^low/−^Langerin^+^CD103^+^ DCs, and that this dermal DC subset was required for priming autoreactive T cells in experimental autoimmune encephalitis (EAE). Here, we compared development of peripheral tissue DCs and susceptibility to EAE in GM-CSF receptor deficient (*Csf2rb* ^−/−^) and *Batf3* ^−/−^ mice. We find that *Batf3*-dependent dermal CD11b^low/−^Langerin^+^ DCs do develop in *Csf2rb* ^−/−^ mice, but that they express reduced, but not absent, levels of CD103. Further, *Batf3* ^−/−^ mice lacking all peripheral CD11b^low/−^ DCs show robust Th cell priming after subcutaneous immunization and are susceptible to EAE. Our results suggest that defective T effector priming and resistance to EAE exhibited by *Csf2rb* ^−/−^ mice does not result from the absence of dermal CD11b^low/−^Langerin^+^CD103^+^ DCs.

## Introduction

GM-CSF regulates the development and activity of several myeloid cell types and influences both the initiation and maintenance of adaptive immune responses [1]. Mice lacking GM-CSF or its receptor show decreased antigen specific T cell priming against the encephalitogenic peptide of myelin oligodendrocyte glycoprotein (MOG_35–55_) and are resistant to EAE [2]. Beyond this role in priming adaptive immune responses, GM-CSF acts to sustain ongoing effector responses, both in EAE initiated by MOG_35–55_ peptide [2] and in collagen-induced arthritis [3]. Similarly, in a murine model of autoimmune myocarditis, GM-CSF acts during priming of Th17 responses by promoting IL-6 and IL-23 production from DCs and during ongoing autoimmune responses by promoting survival of autoreactive CD4^+^ T cells [4].

Several recent studies indicate that it is GM-CSF rather than IL-17 that is the primary Th17-derived cytokine responsible for the development of EAE [5]–[7]. Initially, it was thought that IL-17 production would explain why Th17 cells and RORγt were required for EAE. However, *in vivo* neutralization of IL-17 only reduced EAE severity but did not eliminate disease, and non-IL-17 and non-IFN-γ pathways have been implicated [8]–[10]. IL-23, but not IL-6 or TGF-β, was found to be critical for regulating the pathogenicitiy of Th17 cells [11], [12]. GM-CSF derived from T cells, but not other CNS-resident or peripheral immune cells, was required for development of EAE [5], and acted during the effector phase to augment pathogenicity of Th17 cells [6], [7]. IL-23 induced Th17 cells to increase production of GM-CSF, which further enhanced IL-23 production by antigen-presenting cells [6]. RORγt was shown to induce GM-CSF, which acted on infiltrating myeloid cells entering the CNS, rather than resident microglia, during the development of EAE [7]. These studies suggest a model of EAE in which pathogenic T cells secrete GM-CSF that activates myeloid cells infiltrating the CNS to produce pathogenic lesions and further amplifies IL-23 production by DCs [13].

 A recent report has suggested that the role for GM-CSF in EAE was based on its requirement for the development of dermal CD11b^low/−^Langerin^+^CD103^+^ DCs [14]. Dermal CD11b^low/−^Langerin^+^CD103^+^ DCs represent one anatomic subtype of peripheral tissue CD11b^low/−^CD103^+^ DCs, all of which share with lymphoid tissue CD8α^+^ cDCs developmental dependence on *Batf3*, *Irf8*, and *Id2* [15], [16]. CD8α^+^ cDCs are cross-presenting DCs, characterized by consistent expression of DEC205, and variable expression of CD103 which can be regulated by GM-CSF, IL-3, and TGF-β1 [17]–[19]. Mice lacking GM-CSF or the GM-CSF receptor were reported to lack dermal CD11b^low/−^Langerin^+^CD103^+^ DCs in the skin and peripheral lymph nodes under steady state and inflammatory conditions, and were resistant to EAE [14]. In addition, radiation chimeras expressing the human diphtheria toxin receptor (DTR) controlled by the Langerin promoter [20] and depleted of peripheral CD11b^low/−^Langerin^+^CD103^+^ DCs were resistant to EAE and had reduced priming of MOG_35–55_ -specific T cells [14]. This study concluded that GM-CSF was required for the initiation of EAE because it was required for the development of dermal CD11b^low/−^Langerin^+^CD103^+^ DCs, which were required to prime pathogenic T cells [14].

To test whether loss of GM-CSF receptor protects from EAE by eliminating dermal CD11b^low/−^Langerin^+^CD103+ DCs, we asked if *Batf3* ^−/−^ mice, which also lack this DC subset, are also protected from EAE. We find that *Batf3* ^−/−^ mice develop EAE after immunization and have increased numbers of MOG_35–55_ peptide-specific T cells, excluding a requirement for either peripheral CD11b^low/−^CD103^+^ DCs or lymphoid tissue-resident CD8α^+^ DCs in EAE development. Direct comparison of *Batf3* ^−/−^ and GM-CSF receptor deficient mice revealed that GM-CSF signaling was not required for the development of dermal CD11b^low/−^Langerin^+^CD103^+^ DCs, but rather regulated the expression level of CD103 on all peripheral DCs.

## Results

### *Batf3* ^−/−^ mice develop EAE following MOG immunization

**Figure 1:**
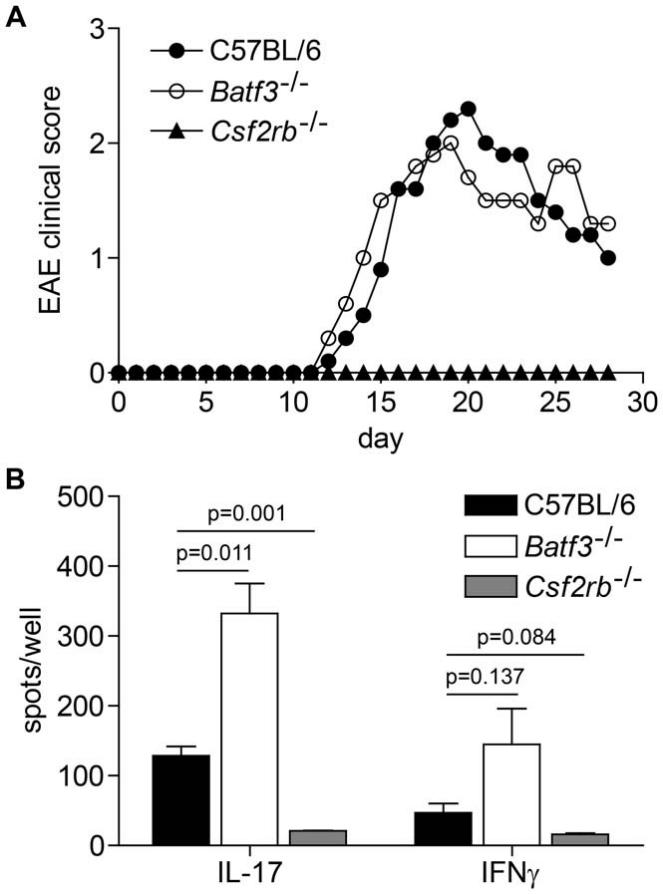
*Batf3* ^−/−^ mice are susceptible to EAE. (A) Clinical course of EAE in C57BL/6 mice, *Batf3* ^−/−^ mice (C57BL/6 background), and *Csf2rb* ^−/−^ mice. Data are combined from two experiments (n = 10 mice per group). Points represent the mean clinical score. For clarity, error bars are not displayed. (B) ELISPOT assays on popliteal lymph node cells at day 7 following footpad immunization (MOG_35–55_ peptide in CFA) and restimulation with MOG_35–55_ peptide. Data are from one of two similar experiments (n = 3 per group). Mean ± SEM, p values by unpaired student's t test.

To compare *Batf3* ^−/−^ mice with mice lacking the GM-CSF receptor (*Csf2rb* ^−/−^) on the same genetic background, we backcrossed *Batf3* ^−/−^ mice onto the C57BL/6 background for 10 generations. *Csf2rb* ^−/−^ mice lack the common β-chain for signaling through receptors for GM-CSF, IL-5, and IL-3. However, mice have a second β-chain, *Csf2rb2*, which selectively pairs with the IL-3 receptor α-chain. Thus, *Csf2rb* ^−/−^ mice lose responsiveness to GM-CSF and IL-5, but not to IL-3 [21], [22]. Wild type (C57BL/6), *Batf3* ^−/−^, and *Csf2rb* ^−/−^ mice were subcutaneously immunized with MOG_35–55_ peptide as previously described and followed for clinical disease [14]. *Csf2rb* ^−/−^ mice remained disease free as reported [14], but *Batf3* ^−/−^ mice developed EAE with the same severity and kinetics as wild type C57BL/6 mice (Fig. 1A).

In draining lymph nodes, examination by ELISPOT assay showed that immunized *Batf3* ^−/−^ mice had an increased frequency of MOG_35–55_-specific T cells producing IL-17 and IFN-γ (Fig. 1B), while *Csf2rb* ^−/−^ mice had a significantly reduced frequency of such cells, as previously reported [14]. Thus, *Batf3* ^−/−^ mice, which lack all peripheral CD11b^low/−^CD103^+^ DCs [16], are able to prime pathogenic CD4^+^ T cells, and remain susceptible to EAE. These findings would disagree with the interpretation that resistance to EAE in *Csf2rb* ^−/−^ mice results from the absence of dermal CD11b^low/−^Langerin^+^CD103^+^ DCs as was recently suggested [14].

### *Csf2rb* ^−/−^ mice retain development of *Batf3*-dependent dermal CD11b^low/−^Langerin^+^ DCs

**Figure 2:**
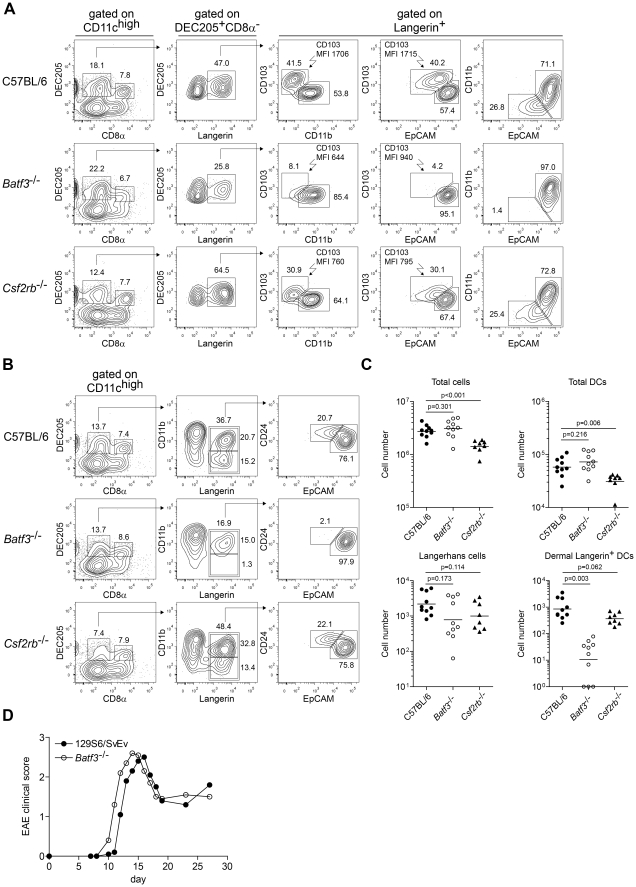
*Batf3*-dependent dermal CD11b^low/−^Langerin^+^ DCs develop in *Csf2rb* ^−/−^ mice, but lack expression of CD103. (A) FACS analysis of SDLN (inguinal) DCs from C57BL/6, *Batf3* ^−/−^ (C57BL/6 background), and *Csf2rb* ^−/−^ mice. Left plots are gated on CD11c^high^ cells. The second column is gated on migratory (DEC205^+^CD8α^−^) DCs. The third, fourth, and fifth columns are gated on Langerin^+^ migratory DCs. Numbers represent the percentage of cells within the indicated gates. MFI are shown for the indicated gates. Data are representative of eight to ten individual inguinal lymph nodes from four to five mice per genotype over two experiments. (B) FACS analysis of SDLN (inguinal) DCs from C57BL/6, *Batf3* ^−/−^, and *Csf2rb* ^−/−^ mice. Left plots are gated on CD11c^high^ cells. Middle plots are gated on migratory (DEC205^+^CD8α^−^) DCs. Right plots are gated on all Langerin^+^ migratory DCs, independent of expression of CD11b. Numbers represent the percentage of cells within the indicated gates. Data are representative of inguinal lymph nodes from at least five mice per genotype for all stains except CD24, which has been performed on one mouse per genotype. (C) Absolute cell numbers per individual inguinal lymph nodes calculated from the FACS analysis in (A). Langerhans cells were gated as Langerin^+^CD11b^+^EpCAM^high^ DCs, and dermal Langerin^+^ DCs were gated as Langerin^+^CD11b^low/−^EpCAM^mid^ DCs. Horizontal bars represent the geometric mean; p values by unpaired student's t test. (D) Clinical course of EAE in 129S6/SvEv mice and *Batf3* ^−/−^ mice (129S6/SvEv background). Data are from one of three similar experiments (n = 5 mice per group). Points represent the mean clinical score. For clarity, error bars are not displayed.

GM-CSF was recently reported to directly regulate CD103 expression on splenic CD8α^+^ DCs [17]–[19], so conceivably *Csf2rb* ^−/−^ mice might retain dermal CD11b^low/−^Langerin^+^ DCs that simply lack CD103. To test this, we examined DCs from C57BL/6, *Batf3* ^−/−^, and *Csf2rb* ^−/−^ mice for expression of markers that distinguish between *Batf3*-dependent and *Batf3*-independent DC subsets (Fig. 2A).

In skin draining lymph nodes (SDLNs), migratory DCs are evident as DEC205^+^CD8α^−^ cells, which can be divided into at least three subsets based on CD11b, Langerin, and CD103 expression [23]–[25]. C57BL/6, *Batf3* ^−/−^, and *Csf2rb* ^−/−^ mice each contained normal populations of dermal Langerin^−^ DCs and Langerhans cells of the epidermis (CD11b^+^Langerin^+^CD103^−^EpCAM^high^) (Fig. 2A). However, differences were evident in the dermal CD11b^low/−^Langerin^+^CD103^+^ DCs between these strains. C57BL/6 mice contained the normal population of dermal CD11b^low/−^Langerin^+^CD103^+^EpCAM^mid^ DCs, which were missing in *Batf3* ^−/−^ mice [16]. Notably, *Csf2rb* ^−/−^ mice retained this population of dermal DCs, but in this strain of mice, these DCs expressed slightly lower levels of CD103 relative to C57BL/6 mice. CD24 can also been used to distinguish between populations of dermal DCs [26], and similarly C57BL/6 mice contained a population of dermal Langerin^+^CD24^+^EpCAM^mid^ DCs that were completely missing in *Batf3* ^−/−^ mice, but which were present in *Csf2rb* ^−/−^ mice (Fig. 2B). *Csf2rb* ^−/−^ mice showed reduced total lymph node cellularity and a reduced number of overall lymph node DCs, but normal numbers of Langerhans cells and dermal CD11b^low/−^Langerin^+^ DCs (Fig. 2C). *Batf3* ^−/−^ mice showed a selective ∼100-fold reduction in the number of dermal CD11b^low/−^Langerin^+^ DCs. These results indicate that *Csf2rb* ^−/−^ mice do contain dermal CD11b^low/−^Langerin^+^ DCs, but that in the absence of GM-CSF signaling, CD103 expression is reduced.

### C57BL/6 *Batf3* ^−/−^ mice contain CD8α^+^ cDCs in SDLNs but not spleen

In analyzing SDLNs of C57BL/6 *Batf3* ^−/−^ mice, we noticed an unexpected population of DEC205^+^CD8α^+^ cDCs that were eliminated in the spleen and SDLNs of 129S6/SvEv *Batf3* ^−/−^ mice [16], [27] (Fig. 2A, 2C). Therefore, we directly compared DCs from SDLNs of *Batf3* ^−/−^ mice on both genetic backgrounds (Fig. S1A). C57BL/6 *Batf3* ^−/−^ mice lacked dermal CD11b^low/−^CD103^+^ DCs (Fig. S1A) but had a persistence of DEC205^+^CD8α^+^ cDCs in peripheral lymph nodes (Fig. S1A, S1B). In spleens, *Batf3* ^−/−^ mice showed a significant decrease in DEC205^+^CD8α^+^ cDCs on both C57BL/6 and 129S6/SvEv backgrounds compared to controls (Fig. S1C, S1D). On the 129S6/SvEv background, DEC205^+^CD8α^+^ cDCs were reduced from 4.8% of splenic cDCs in control mice to only 0.11% of splenic cDCs in *Batf3* ^−/−^ mice. On the C57BL/6 background, DEC205^+^CD8α^+^ cDCs were reduced from 14% of splenic cDCs in control mice to only 2.5% of splenic cDCs in *Batf3* ^−/−^ mice (Fig. S1C, S1D). Thus, for splenic cDCs, *Batf3* is non-redundant in the development of DEC205^+^CD8α^+^ cDCs.

While loss of *Batf3* does not eliminate the population of DEC205^+^CD8α^+^ cDCs in SDLNs, these cells did not express CD103 normally (Fig. S1E). 9% of the DEC205^+^CD8α^+^ cDCs in SDLNs of control mice expressed CD103, while only 1.8% expressed CD103 from *Batf3* ^−/−^ mice. Notably, CD103 expres sion was also reduced on this population from SDLNs of *Csf2rb* ^−/−^mice. Conceivably, the persistence of DEC205^+^CD8α^+^ cDCs in SDLNs on the C57BL/6 background could result from genetic polymorphisms that impact the redundancy of *Batf3* in the development of these cells. Alternately, variable inflammation between strains could be involved, since infection with *Toxoplasma gondii* can regulate the size of splenic CD11b^low/−^CD103^+^ DC populations [28].

### 129S6/SvEv *Batf3* ^−/−^ are susceptible to EAE

Conceivably, persistence of DEC205^+^CD8α^+^ cDCs in SDLNs of C57BL/6 *Batf3* ^−/−^ mice was the basis for their susceptibility to EAE, based on potentially overlapping functions for DEC205^+^CD8α^+^ cDCs and dermal CD11b^low/−^CD103^+^ DCs [29]. To test this, we examined *Batf3* ^−/−^ mice on the 129S6/SvEv background, which lack both DEC205^+^CD8α^+^ and dermal CD11b^low/−^CD103^+^ DCs in both spleen and SDLNs (Fig. 2D). As with C57BL/6 *Batf3* ^−/−^ mice, *Batf3* ^−/−^ mice on the129S6/SvEv background were equally as susceptible to EAE as wild type controls. Thus, loss of dermal CD11b^low/−^CD103^+^ DCs could not have explained the resistance to EAE that was observed in *Csf2rb* ^−/−^ mice.

### *Csf2rb* ^−/−^ mice retain *Batf3*-dependent CD11b^low/−^ peripheral tissue DCs with reduced CD103 expression

**Figure 3:**
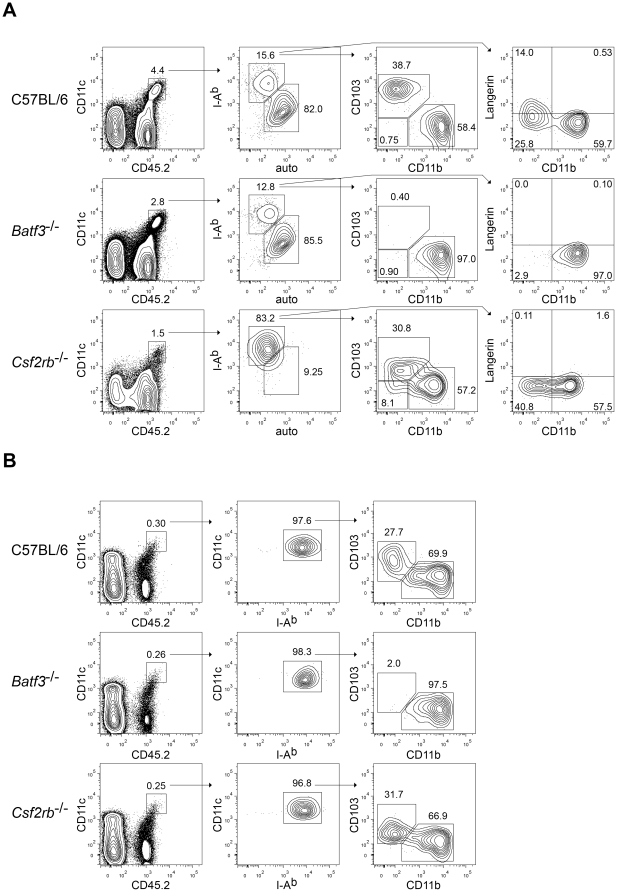
GM-CSF controls the level of CD103 expression on *Batf3*-dependent CD11b^low/−^ peripheral DCs. (A) FACS analysis of lung DCs from C57BL/6, *Batf3* ^−/−^ (C57BL/6 background), and *Csf2rb* ^−/−^ mice. Left plots are gated on live, single cells. The second column of plots is gated on CD11c^+^CD45.2^+^ cells, consisting of a mixture of lung macrophages and DCs. Note that in *Csf2rb* ^−/−^ mice, lung macrophages lack CD11c expression [38], and therefore this gate includes only DCs. The third and fourth columns of plots are gated on I-A^bhigh^ autofluorescence^low^ DCs. Numbers represent the percentage of cells within the indicated gates. Data are representative of six individual lungs from three mice per genotype over two experiments. (B) FACS analysis of kidney DCs from C57BL/6, *Batf3* ^−/−^ (C57BL/6 background), and *Csf2rb* ^−/−^ mice. Left plots are gated on live, single cells. The second column of plots is gated on CD11c^high^CD45.2^+^ cells, consisting of DCs. The third column of plots is gated on CD11c^high^I-A^b+^ DCs. Numbers represent the percentage of cells within the indicated gates. Data are representative of six individual kidneys from three mice per genotype over two experiments.

We next compared C57BL/6, *Batf3* ^−/−^, and *Csf2rb* ^−/−^ mice for the presence of DC subsets in peripheral tissues, specifically the lung and kidney. In the lung, DCs exist as two populations, characterized as *Batf3*-dependent CD11b^low/−^Langerin^mid^CD103^+^ DCs and *Batf3*-independent CD11b^+^Langerin^−^CD103^−^ DCs. Lungs from *Batf3* ^−/−^ mice completely lacked CD11b^low/−^Langerin^mid^CD103^+^ DCs (Figure 3A) as expected [16]. Importantly, lungs from *Csf2rb* ^−/−^ mice retained a normal population of pulmonary CD11b^low/−^ DCs, which expressed reduced levels of CD103 and undetectable levels of Langerin (Fig. 3A). *Csf2rb* ^−/−^ mice develop alveolar proteinosis due to defective surfactant handling by lung macrophages in the absence of GM-CSF signaling [21]. Conceivably, this inflammatory process could have influenced CD103 expression by CD11b^low/−^ lung DCs. To test this, we examined CD103 expression by DCs of the kidney, since this organ shows no pathology in *Csf2rb* ^−/−^ mice. Again, while *Batf3* ^−/−^ mice completely lacked CD11b^low/−^CD103^+^ kidney DCs, *Csf2rb* ^−/−^ mice had normal numbers of CD11b^low/−^ kidney DCs, but which expressed moderately reduced levels of CD103 (Fig. 3B). In summary, it appears that *Csf2rb* ^−/−^ mice retain normal development of peripheral tissue CD11b^low/−^ which express reduced levels of CD103.

### Basal CD103 expression by CD8α^+^-equivalent cDCs requires *Batf3* but not GM-CSF signaling

**Figure 4:**
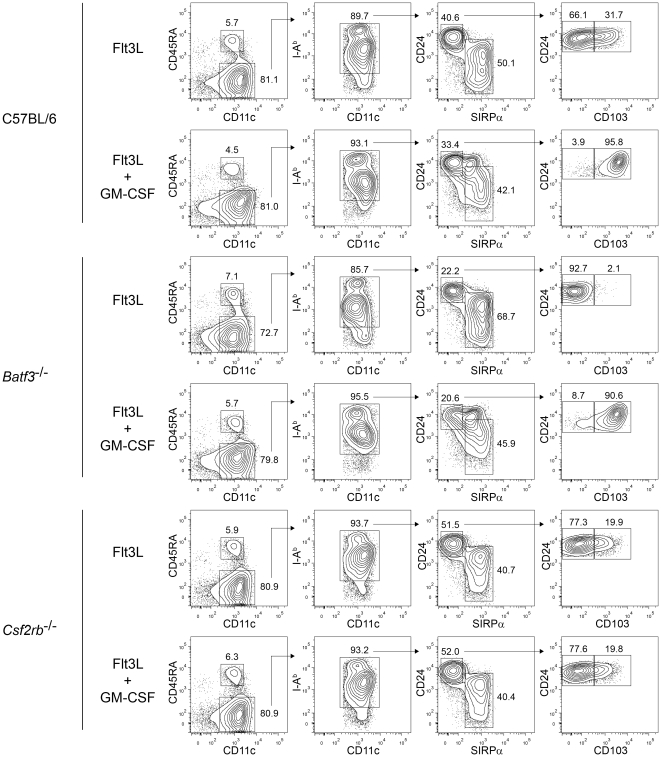
GM-CSF induces CD103 expression on Flt3L-derived CD8α-equivalent DCs. FACS analysis of developing DCs in bone marrow cells from C57BL/6, *Batf3* ^−/−^ (C57BL/6 background), and *Csf2rb* ^−/−^ mice cultured with Flt3L for 9 days, with or without the addition of GM-CSF to the culture on day 7. The left column is gated on live single cells, the second on CD11c^+^CD45RA^−^ cDCs, the third on I-A^b+^ cDCs, and the fourth on CD24^high^Sirpα^low^ cells. Numbers represent the percentage of cells within the indicated gates. Data are representative of bone marrow cells from two mice per genotype over two experiments.

While CD103 has been used as a marker for the dermal CD11b^low/−^Langerin^+^ DC lineage [14], recent work shows that CD103 expression can be dynamically regulated by cytokines [17], [18]. Therefore, we wished to re-examine the relationships between CD103 expression, *Batf3*, and GM-CSF signaling. We selected the Flt3L-induced DC differentiation from bone marrow cultures for this purpose since it has allowed the analysis of CD103 expression as dependent on both *Batf3* and GM-CSF [17], [18] (Fig. 4).

Previously, CD24^high^ SIRPα^low^ DCs were considered an “equivalent” of CD8α^+^ cDCs in Flt3L-treated bone marrow cultures [30], despite the fact that only a subpopulation of these cells express CD103 (Fig. 4). The CD103^+^ fraction of CD24^high^ SIRPα^low^ DCs was more potent than the CD103^−^ fraction for cross-presentation [17], suggesting a correlation between CD103 expression and CD8α^+^ cDC function. We find that addition of GM-CSF during day 7 through 9 of Flt3L culture induces CD103 expression on all CD24^high^ SIRPα^low^ DCs (Fig. 4), in agreement with recent reports which showed that such GM-CSF-induced cells exhibit robust cross-presentation [17], [18]. However, while addition of IL-3 and TGF-β1 to Flt3L cultures can both induce expression of CD103, only IL-3 induced the capacity for cross-presentation [17], dissociating CD103 expression from cross-presentation. Likewise, we found that GM-CSF treatment induced CD103 expression on all CD24^high^SIRPα^low^ DCs, even in bone marrow cultures from *Batf3* ^−/−^ mice (Fig. 4), which completely lack CD103^+^ DCs *in vivo* [16], further dissociating CD103 expression from the properties of true CD8α^+^ cDC equivalents. In agreement, a recent report found that GM-CSF-induced CD103^+^CD24^high^SIRPα^low^ Flt3L-derived DCs from *Batf3* ^−/−^ mice lacked the capacity for cross-presentation [18]. Finally, *Csf2rb* ^−/−^ bone marrow generated a subpopulation of CD103^+^CD24^high^SIRPα^low^ DCs, indicating development that is independent of GM-CSF signaling, although whether these arise from the actions of cytokines such as IL-3, or arise independently of soluble factors present in the culture remains unknown. In summary, these results indicate that CD103 expression can be regulated by GM-CSF, independently of *Batf3*, and may not represent a static marker of *Batf3*-dependent DC lineages.

## Discussion

 Our study makes two new observations. First, we show that CD11b^low/−^ non-lymphoid DCs develop in the absence of GM-CSF signaling. Second, we show that *Batf3*-dependent CD11b^low/−^ non-lymphoid DCs are not required for T effector cell priming or clinical EAE after subcutaneous immunization. King et al. recently concluded that mice deficient for GM-CSF or GM-CSF receptor were resistant to EAE because they lacked dermal CD11b^low/−^Langerin^+^CD103^+^ DCs, which they proposed were required for priming effector CD4 T cells with MOG_35–55_ [14]. Dermal CD11b^low/−^Langerin^+^CD103^+^ DCs are a conventional DC subset that requires the transcription factor *Batf3* for their development [16]. Here, we directly compared *Csf2rb* ^−/−^ with *Batf3* ^−/−^ mice for their susceptibility to EAE and for their development of various DC subsets using a broad panel of DC markers, including Langerin, CD11b, EpCAM, CD24, and CD103. We confirm that the dermal CD11b^low/−^Langerin^+^CD103^+^ DC subset is absent in *Batf3* ^−/−^ mice, but surprisingly find that *Csf2rb* ^−/−^ mice harbor normal numbers of this DC subset in SDLNs as a CD11b^low/−^ Langerin^+^EpCAM^mid^CD24^high^ cell that expresses reduced, but not absent, levels of CD103. Moreover, in other peripheral tissues, *Csf2rb* ^−/−^ mice have similar CD11b^low/−^CD103^+^ DCs that express reduced, but not absent, levels of CD103.

Our data agree with recent reports that GM-CSF directly regulates CD103 expression on Flt3L-derived CD8α^+^ equivalent cDCs and splenic DEC205^+^CD8α^+^ cDCs [17]–[19]. Beyond this, we show that GM-CSF also regulates levels of CD103 expression on peripheral tissue-resident CD11b^low/−^ DCs, which were not examined in the previous studies. Notably, a separate report has suggested that GM-CSF signaling is required for the development of small intestinal lamina propria CD11b^+^CD103^+^ DCs [31], although their data could be reinterpreted as simply representing reduced CD103 expression by these DCs. In that report, *Csf2rb* ^−/−^mice had half as many lamina propria CD11b^+^CD103^+^ DCs as wild type mice, but 1.7-fold more CD11b^+^CD103^−^ DCs than wild type mice, consistent with a reduction of CD103 expression as an explanation for the “missing” subset.

 King et al. also found decreased T cell priming to subcutaneous immunization with MOG_35–55_ peptide and partial EAE resistance after *in vivo* depletion of peripheral Langerin^+^CD103^+^ DCs using radiation chimeras reconstituted with bone marrow from transgenic mice expressing DTR under the control of the Langerin promoter [20]. The use of radiation chimeras allowed the selective depletion of dermal CD11b^low/−^Langerin^+^ DCs, avoiding depletion of epidermal CD11b^+^Langerin^+^ Langerhans cells, because the latter are radiation-resistant, and were therefore of host origin and did not express the DTR. Our results indicate that *Csf2rb* ^−/−^ mice are not deficient in dermal CD11b^low/−^Langerin^+^ DCs, but rather that these cells have reduced CD103 expression. However, *Batf3* ^−/−^ mice lack this dermal DC subset, and yet are susceptible to EAE induction and display increased, not decreased, T cell priming to MOG_35–55_ peptide immunization. It is unclear why DTR-mediated depletion of donor-derived, radiation-sensitive Langerin^+^ DCs reduces priming and delays EAE, when genetic ablation of these cells in *Batf3* ^−/−^ mice leaves priming and EAE intact. Their use of DTR-mediated depletion involved irradiation of mice prior to immunization, which may have had unintended consequences. Furthermore, a study by Henri et al. has identified additional dermal DC subsets [26], including one that is Langerin^+^ but CD103^−^, although it's *Batf3*-dependence was not characterized. Conceivably, the depletion of this subset by diphtheria toxin treatment could contribute to differences between our results and those of King et al. Notably, DTR-mediated depletion reduced the severity of EAE, but with a profound decrease only in MOG_35–55_-specific IFN-γ-secreting T cells, with minimal change in the number of IL-17-secreting T cells. In contrast, MOG_35–55_-immunized *Csf2rb* ^−/−^ mice had profound reductions in antigen-specific IFN-γ-secreting and in IL-17-secreting T cells, suggesting that *Csf2rb* ^−/−^ mice differ from mice in which Langerin^+^ DCs are depleted using DTR.

 Our results suggest that the absence of dermal CD11b^low/−^Langerin^+^CD103^+^ DCs leads to augmented T cell priming of both Th17 and Th1 responses. Igyártó et al. have recently shown that skin infection of *Batf3* ^−/−^ mice with recombinant *Candida albicans* led to reduced Th1 but increased Th17 responses compared to control mice [32]. These authors demonstrated that dermal Langerin^+^CD103^+^ DCs expressed IL-12, explaining their ability to promote Th1 cell responses, and IL-27, a cytokine known to inhibit Th17 cell responses [33], [34]. While we found increased Th17 responses in *Batf3* ^−/−^ mice immunized with MOG_35–55_ and CFA, we also observed increased Th1 responses compared to control mice. Conceivably, the different Th1 responses between our study and Igyártó et al. could arise from differences in the form of immunization, relying on CFA versus fungal infection. Notably, blood-derived inflammatory monocytes have been reported to serve as a critical source of IL-12 after CFA-based subcutaneous immunization [35]. While we observed between a 2 to 3-fold higher frequency of IL-17-producing T cells in MOG_35–55_-immunized *Batf3* ^−/−^ mice relative to controls, the clinical EAE scores were similar between these groups. Conceivably, the frequency of IL-17-producing T cells is unrelated to the severity of EAE when Th17 cells are above some threshold frequency. Alternatively, IL-17 itself may not be the limiting factor in regulating the intensity of EAE, since GM-CSF also recently was implicated as the major Th17-derived cytokine responsible for driving pathogenic lesions in this model [5]–[7]. In fact, however, we do observe a slightly more rapid onset of EAE in *Batf3* ^−/−^ mice, which was more pronounced in the 129S6/SvEv genetic background (Fig. 2D), which could relate to increased Th17 development.

If a lack of peripheral CD11b^low/−^ DCs does not abrogate priming of T effector cells after subcutaneous immunization, why then does this priming defect occur in *Csf2rb* ^−/−^ mice? One possibility is that other DC subsets besides dermal CD11b^low/−^ DCs can prime CD4 T cells [35], but that the ability of these other DCs to prime requires that they receive a GM-CSF signal. Indeed, GM-CSF is essential for IL-6 and IL-23 production by DCs during the priming phase of autoimmune myocarditis [4] and is required for inflammatory DC development from monocytes in a model of repeated immunization with methylated BSA and CFA [36]. T cells do not express GM-CSF receptor, so an intrinsic T cell defect seems unlikely. While the source of GM-CSF after subcutaneaous immunization is still unclear, antigen-specific T cells can produce GM-CSF [5]–[7] and may augment responses by DCs during initial priming of T effector cells.

## Materials and Methods

### Ethics statement

This study was carried out in strict accordance with the recommendations in the Guide for the Care and Use of Laboratory Animals of the National Institutes of Health. The protocol was approved by the Animal Studies Committee of Washington University (#20090320).

### Mice

Wild-type 129S6/SvEv and C57BL/6 mice were purchased from Taconic. *Batf3* ^−/−^ mice on a 129S6/SvEv background were previously generated in our laboratory [27], and were backcrossed for 10 generations to the C57BL/6 background. *Csf2rb* ^−/−^ mice on the C57BL/6 background were purchased from Jackson Laboratory. Experiments were performed with sex-matched mice at 8–20 weeks of age. Mice were bred and maintained in our specific pathogen-free animal facility according to institutional guidelines.

### Induction of EAE

EAE was induced as previously described [14]. Briefly, mice were immunized subcutaneously with 100 micrograms MOG_35–55_ peptide (Sigma Genosys) emulsified in complete Freund's adjuvant (CFA) (made with 5 mg/ml heat-killed *Mycobacterium tuberculosis* H37Ra (BD Difco) in incomplete Freund's adjuvant (BD Difco)). Pertussis toxin (List Biological Laboratories) was injected intraperitoneally (300 ng) on days zero and two. Mice were observed for signs of EAE and graded on a standard 0–5 scale as described [37].

### T cell priming and ELISPOT assays

Mice were immunized subcutaneously in the hind footpads with 10 nanomoles MOG_35–55_ peptide (Sigma Genosys) emulsified in complete Freund's adjuvant (CFA) (BD Difco). Popliteal lymph nodes were collected at day 7, and single cell suspensions were used in ELISPOT assays with the IFNγ ELISPOT antibody pair from BD Biosciences on Multiscreen Filter Plates from Millipore. An IL-17 ELISPOT was designed using anti-IL17A capture (clone TC11-18H10) and detection (clone TC11-8H4.1) antibodies (BD Biosciences). Cells were plated at 1×10^6^ cells/well, in duplicate, and stimulated with either no antigen, or 10 micromolar MOG_35–55_ peptide. Plates were developed after 16 hours of stimulation at 37°C, and spots were counted on an Immunospot counter (Cellular Technology Ltd.).

### Antibodies

The following antibodies were purchased from BD Biosciences: PE-Cy7 anti-CD11b (M1/70), PerCP-Cy5.5 anti-CD8α (53-6.7), V500 anti-CD8α (53-6.7), PE anti-CD45RA (14.8), biotin anti-CD24 (30F1), FITC CD24 (M1/69), APC anti-CD172a/SIRPα (P84). FITC anti-CD11b (M1/70), APC eFluor 780-anti-CD11c (N418), PE anti-CD103 (2E7), biotin anti-CD103 (2E7), eFluor450 anti-CD317/BST2 (eBio927), eFluor450 anti-MHCII (I-A/I-E) (M5/114.15.2), APC anti-CD45.2 (104), and streptavidin-eFluor450 were purchased from eBioscience. PE anti-CD205/DEC205 (NLDC-145) and APC anti-CD205/DEC205 (NLDC-145) were purchased from Miltenyi. Alexa Fluor 488 anti-CD207/Langerin (929F3.01) was purchased from Imgenex. PE anti-CD326/EpCAM (G8.8) was purchased from Biolegend.

### Flow cytometry

Inguinal lymph nodes and spleens were minced and digested in 5 ml Iscove's modified Dulbecco's media +10% FCS (cIMDM) with 250 µg/ml collagenase B (Roche) and 30 U/ml DNase I (Sigma-Aldrich) for 1 h at 37°C with stirring. Lungs and kidneys were perfused with 10 ml DPBS via injection into the right ventricle after transection of the lower aorta. Dissected lungs and kidneys were minced and digested in 5 ml of cIMDM with 4 mg/ml collagenase D (Roche) for 1 h at 37°C with stirring. EDTA (5 mM final concentration) was added to cell suspensions, and cells were incubated on ice for 5 min. Cells were passed through an 80 µm strainer before red blood cells lysis with ACK lysis buffer. Cells were counted on a Vi-CELL analyzer, and 2–5×10^6^ cells were used per antibody staining reaction.

Staining was performed at 4°C in the presence of Fc Block (clone 2.4G2, BD Biosciences or BioXcell) in FACS buffer (DPBS+0.5% BSA+2 mm EDTA+0.02% sodium azide). For experiments involving intracellular anti-Langerin staining, cells were surface antibody stained prior to fixation in 4% formaldehyde (Thermo Scientific) for 15 minutes at room temperature. Cells were subsequently permeabilized (DPBS+0.1% BSA+0.5% saponin) for 5 min at 4°C and stained in the same permeabilization buffer with anti-Langerin antibody. Cells were washed twice in permeabilization buffer before returning them to FACS buffer. Flow cytometry was performed on a FACSCantoII (BD Biosciences), and data was analyzed with FlowJo software (Tree Star, Inc.).

### Bone marrow cultures

Bone marrow cells from femurs and tibias were collected and red blood cells were lysed in ACK lysis buffer. Cells were cultured in four ml cIMDM in six-well plates at 2×10^6^ cells/ml containing 160 ng/ml murine Flt3L (Peprotech). At day seven of culture, murine GM-CSF (Peprotech) was added to some wells at 20 ng/ml. Non-adherent cells were collected at day nine for flow cytometry.

## Supporting Information

Figure S1 **Flow cytometric analyses of DCs in** ***Batf3*** **^−/−^ mice.** (A) FACS analysis of SDLN (inguinal) DCs from *Batf3* ^+/+^ and *Batf3* ^−/−^ mice on both the C57BL/6 and 129S6/SvEv backgrounds. Left plots from mice of each background are gated on CD11c^+^BST2^−^ cDCs. Right plots from mice of each background are gated on migratory (DEC205^+^CD8α^−^) DCs. Numbers represent the percentage of cells within the indicated gates. Data are representative of at least six mice per genotype and background obtained in several independent experiments. (B) Absolute cell numbers of DEC205^+^CD8α^+^ DCs per individual inguinal lymph nodes from C57BL/6 and *Batf3* ^−/−^ mice (C57BL/6 background) calculated from the FACS analysis performed in Figure 2A. Horizontal bars represent the geometric mean, p value by unpaired student's t test. (C) and (D) FACS analysis of splenic DCs from *Batf3* ^+/+^ and *Batf3* ^−/−^ mice on both the (C) C57BL/6 and (D) 129S6/SvEv backgrounds. Plots are gated on CD11c^+^I-A^b+^ cDCs. Numbers represent the percentage of cells within the indicated gates. Data are representative of at least six mice per genotype and background obtained in several experiments. (E) FACS analysis of SDLN (inguinal) DCs from C57BL/6, *Batf3* ^−/−^ (C57BL/6 background), and *Csf2rb* ^−/−^ mice. Left plots are gated on CD11c^high^ cells. Right plots are gated on DEC205^+^CD8α^+^ cDCs. Numbers represent the percentage of cells within the indicated gates. Data are representative of eight to ten individual inguinal lymph nodes from four to five mice per genotype obtained in two experiments. Data is depicted in these plots are derived from analysis of the same mice used in Figure 2A, but gated to analyze CD103 expression on DEC205^+^CD8α^+^ cDCs.
(TIF)
