# Supplementary figures and images for: Batf3-Dependent CD11blow/− Peripheral Dendritic Cells Are GM-CSF-Independent and Are Not Required for Th Cell Priming after Subcutaneous Immunization
Source: PLoS One. 2011 Oct 17;6(10):e25660. doi: 10.1371/journal.pone.0025660 (PMC3196467; doi:10.1371/journal.pone.0025660)

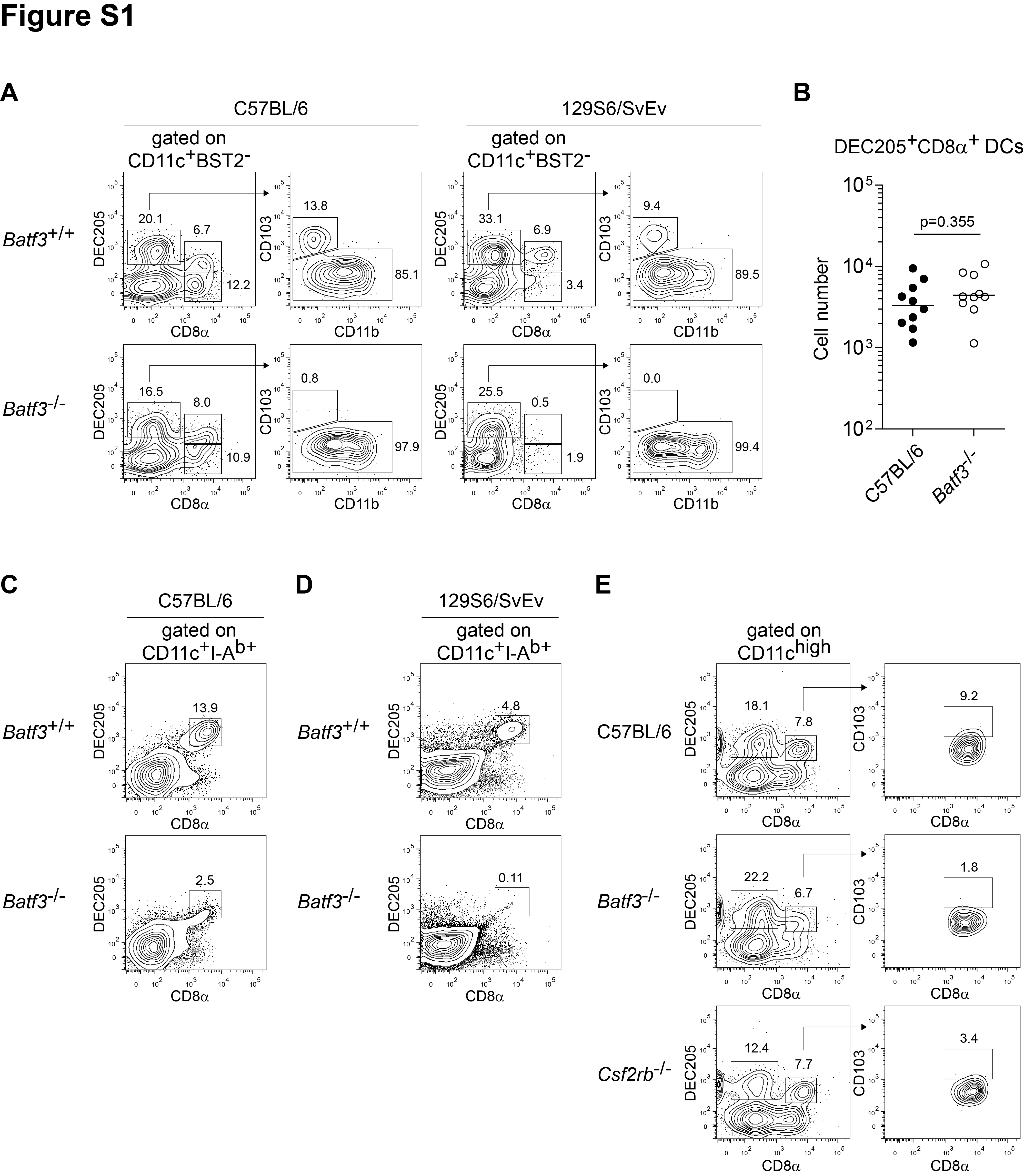

Supplement: Figure S1 — Flow cytometric analyses of DCs in Batf3 −/− mice. (A) FACS analysis of SDLN (inguinal) DCs from Batf3 +/+ and Batf3 −/− mice on both the C57BL/6 and 129S6/SvEv backgrounds. Left plots from mice of each background are gated on CD11c+BST2− cDCs. Right plots from mice of each background are gated on migratory (DEC205+CD8α−) DCs. Numbers represent the percentage of cells within the indicated gates. Data are representative of at least six mice per genotype and background obtained in several independent experiments. (B) Absolute cell numbers of DEC205+CD8α+ DCs per individual inguinal lymph nodes from C57BL/6 and Batf3 −/− mice (C57BL/6 background) calculated from the FACS analysis performed in Figure 2A. Horizontal bars represent the geometric mean, p value by unpaired student's t test. (C) and (D) FACS analysis of splenic DCs from Batf3 +/+ and Batf3 −/− mice on both the (C) C57BL/6 and (D) 129S6/SvEv backgrounds. Plots are gated on CD11c+I-Ab+ cDCs. Numbers represent the percentage of cells within the indicated gates. Data are representative of at least six mice per genotype and background obtained in several experiments. (E) FACS analysis of SDLN (inguinal) DCs from C57BL/6, Batf3 −/− (C57BL/6 background), and Csf2rb −/− mice. Left plots are gated on CD11chigh cells. Right plots are gated on DEC205+CD8α+ cDCs. Numbers represent the percentage of cells within the indicated gates. Data are representative of eight to ten individual inguinal lymph nodes from four to five mice per genotype obtained in two experiments. Data is depicted in these plots are derived from analysis of the same mice used in Figure 2A, but gated to analyze CD103 expression on DEC205+CD8α+ cDCs. (TIF) [file pone.0025660.s001.tif]
